# Th1 cytokines sensitize HER-expressing breast cancer cells to lapatinib

**DOI:** 10.1371/journal.pone.0210209

**Published:** 2019-01-18

**Authors:** Loral E. Showalter, Crystal Oechsle, Nirmala Ghimirey, Chase Steele, Brian J. Czerniecki, Gary K. Koski

**Affiliations:** 1 Department of Biological Sciences, Kent State University, Kent, OH, United States of America; 2 Department of Breast Oncology, Moffitt Cancer Center, Tampa, FL, United States of America; University of South Alabama Mitchell Cancer Institute, UNITED STATES

## Abstract

The HER family of receptor tyrosine kinases has been linked to deregulation of growth and proliferation for multiple types of cancer. Members have therefore become thefocus of many drug and immune-based therapy innovations. The targeted anti-cancer agent, lapatinib, is a small molecule inhibitor that directly interferes with EGFR (HER-1)and HER-2 signaling, and indirectly reduces HER-3 signaling, thus suppressing important downstream events. A recently-developed dendritic cell-based vaccine against early breast cancer (ductal carcinoma in situ; DCIS) that generates strong Th1-dominated immunity against HER-2 has induced pathologic complete response in about one-third of immunized individuals. In vitro studies suggested cytokines secreted by Th1 cells could be major contributors to the vaccine effects including induction of apoptosis and suppression of HER expression. With a view toward improving complete response rates, we investigated whether the principle Th1 cytokines (IFN-γ and TNF-α) could act in concert with lapatinib to suppress activity of breast cancer lines in vitro. Lapatinib-sensitive SKBR3, MDA-MB-468 and BT474 cells were incubated with Th1 cytokines, lapatinib, or both. It was found that combined treatment maximized metabolic suppression(Alamar Blue assay), as well as cell death (Trypan Blue) and apoptosis(Annexin V/Propidium Iodide and TMRE staining). Combined drug plus cytokine treatment also maximized suppression of both total and phosphorylated forms of HER-2 and HER-3. Interestingly, when lapatinib resistant lines MDA-MB-453 and JIMT-1 were tested, it was found that the presence of Th1 cytokines appeared to enhance sensitivity for lapatinib-induced metabolic suppression and induction of apoptotic cell death, nearly abrogating drug resistance. These studies provide pre-clinical data suggesting the possibility that targeted drug therapy may be combined with vaccination to enhance anti-cancer effects, and furthermore that robust immunity in the form of secreted Th1 cytokines may have the capacity to mitigate resistance to targeted drugs.

## Introduction

Breast cancer exists as a public health crisis throughout the world with about 1.4 million cases of invasive breast cancer (IBC) recorded yearly, leading to approximately 500,000 deaths [1]. The United States National Cancer Institute estimated in 2006 that national direct expenditures for breast cancer were valued at over 13 billion dollars [2]. These costs represent an almost unbearable burden for both our health care system, as well as thevictims of breast cancer who must endure the financial and personal costs associated with breast cancer treatment. Clearly new and better approaches are needed to improve the lives of women diagnosed with breast cancer.

To this end, we have developed a vaccine platform based on peptide-loaded IL-12-secreting autologous dendritic cells that generates strong and durable Th1 immunity against the HER-2 oncodriver [3–5]. When used in the neoadjuvant setting to vaccinate subjects with HER-2^pos^ ductal carcinoma in situ of the breast (DCIS), it was found that approximately 18% of the women had no evidence of remaining disease at the time of surgery (pathologic complete response; pCR). Furthermore, for about half of the women with residual disease, HER-2 expression levels were strongly suppressed [3, 4]. In addition, immunohistochemical studies revealed heavy infiltrates of both CD4^pos^ T cells and CD20^pos^ B cells to the areas of disease, but relatively fewer CD8^pos^ T cells, suggesting a central role for helper T cells in anti-tumor immunity [3, 4]. Indeed, in follow-onstudies, we demonstrated that the paired combination of the defining Th1 cytokines, IFN-γ and TNF-α, could mediate in vitro many of the effects observed in vaccinated individuals including significant suppression of HER-family RTK surface expression and induced apoptotic cell death in HER family-expressing breast cancer cell lines [6]. These latter studies, demonstrating the potency of multiplexed Th1 cytokines, offer a consistent explanation of how CD4^pos^Th cells, which cannot recognize tumor cells directly, may nonetheless play a decisive role in their elimination.

An idealized vaccine or other immunotherapy holds several potential advantages compared with the standard interventions of surgery, radiation and chemotherapy. Chief among these is the promise of a treatment with fewer harsh side-effects and associated morbidities the current modalities entail. So while the realization of a Th1-polarizing vaccine that acts in concert with standard chemo/trastuzumabtherapy to improve outcomes would be a highly welcome addition to our armamentarium, it would be better still to avoid traditional chemotherapy and pair vaccination with targeted agents, preferably small-molecule drugs with the lowest possible toxicity profiles. This approach has been recently validated by our group. Our original DCIS vaccine trial targeting HER-2 showed that only around 5% of patients with estrogen receptor (ER)-expressing tumors experienced pCR to neoadjuvant vaccination, while about 40% of ER^neg^ patients had pCRs [4]. We demonstrated in vitro that ER^pos^/HER-2^pos^ breast cancer lines were less susceptible to Th1 cytokines than their ER^neg^/HER-2^pos^ counterparts, and this disparity in sensitivity could be ablated by the addition of anti-estrogen drugs coincident with cytokine exposure. This observation was used as the basis for a new clinical trial where a short course of anti-estrogen drugs was given for ER^pos^/HER-2^pos^DCIS subjects during the 6-week period of vaccination. This simple modification raised ER^pos^ pCR levels from 5% to 30%, so that there was no longer a statistically-significant difference in pCR rates between ER^pos^ and ER^neg^ subjects [7]. These studies therefore supported the concept that targeted drugs with lower toxicity profiles than standard cytotoxic chemotherapy could dramatically leverage vaccine responses. They also appeared to validate our in vitro screening approach where candidate drugs are tested and selected for their ability to amplify Th1 cytokine-mediated killing of HER-2^pos^ breast cancer lines as a prelude to their being tested in clinical trials.

The next logical step to further improve vaccine efficacy would be to identify additional targeted drugs that will work synergistically, or at least additively with Th1 cytokines to enhance apoptosis in breast cancer cell lines. Such drugs could then be rapidly translated into clinical trials (as we have done with anti-estrogen drugs) to test for their ability to enhance vaccine effects. Lapatinib (Tykerb) is a small-molecule inhibitor of kinase activity for both EGFR (HER-1) and HER-2 [8, 9] that has found use in breast cancer treatment in combination with chemotherapeutic agents [10, 11] and with the HER-2-directed monoclonal antibody drug trastuzumab [12, 13]. A possible advantage of lapatinib over trastuzumabis its ability to cross the blood brain barrier to gain access to CNS metastases of HER-2^pos^ breast cancer [14], in addition to lower reported incidence of cardiotoxicity [15, 16]. It should be noted, however, that a drawback of lapatinib is its associated GI toxicity [17, 18]. In the studies presented here we show that lapatinib is indeed capable of enhancing Th1 cytokine-induced apoptosis of HER-2^pos^ breast cancer lines thus providing justification for testing this drug in conjunction with Th1-polarizing dendritic cell vaccines for enhancement of anti-tumor activity.Also demonstrated is the surprising observation that the presence of Th1 cytokines appears to re-sensitize lapatinib-resistant cell lines to the drug.

## Material and methods

### Cell culture

SK-BR-3, BT-474, MDA-MD-468, MDA-MB-453 and HCC1419 cell lines were obtained from American Type Culture Collection (Rockwell, MA).The JIMT-1 cells were a gift from Pravin Kaumaya, (The Ohio State University, Columbus OH). SKBR3 cells were cultured in McCoy’s 5A Media (Gibco) supplemented with 10% v/v fetal calf serum (FBS; BioWhittaker), 100 units/ml of potassium penicillin and 100 μg/ml of streptomycin sulfate (BioWhittaker). BT-474 cells were cultured in HybriCare (ATCC), supplemented with 10% v/v FBS, 100 units/ml of potassium penicillin and 100 ug/ml of streptomycin sulfate. MDA-MB-468, MDA-MB-453 and HCC1419 cells were cultured in RPMI-1640 (BioWhittaker), 10% v/v FBS, 100 units/ml of potassium penicillin and 100 ug/ml of streptomycin sulfate (BioWhittaker), 2mmol/L glutamine (BioWhittaker), 1mmol/L sodium pyruvate (BioWhittaker), and 1% non-essential amino acids (BioWhittaker). All cells were maintained in culture at 37°C in 5% CO_2_.

### Alamarblue assay

Cellular metabolism (consistent with cell viability) was measured by theAlamar Blue assay. Breast cancer cell lines were harvested using trypsin (Lonza), counted and plated at a density of approximately 5x10^4^ cells per well in 48-well cluster plates containing 500μl culture media. Cells were treated the next day with 20 ng/ml TNF-α (Peprotech), 12.5 ng/ml of IFN-γ (Peprotech), or 0.2μM lapatinib and incubated for approximately 72 hours. On day 3, 5μl of 56mM stock resazurin sodium salt was added to each well to achieve a final concentration of 560μM. The plates were then further incubateduntil color change was evident. The reduction of resazurin to resorufin was measured via optical density at 630nm with a Bio-Tek ELx800 absorbance reader.

### Trypan blue exclusion assay

Cells were plated and treated in a manner identical to that described for the Alamar Blue assay. On day 3, cells were harvested by scraping, pelleted at 218g for 5 minutes, and resuspended. Live/dead staining using the Trypan Blue dye was assessed by two methods. The first was the traditional microscopy-based technique where cells were resuspended in a 1:1 solution of PBS and Trypan Blue. The cells were then counted on a hemocytometer and determined to be dead if they retained stain and alive if they excluded the dye. The second method took advantage of the fluorescent properties of the Trypan Blue dye, and was flow cytometry-based. Here, the resuspended cells were resuspendedina 0.002% (w/v) solution of trypan blue in PBS. Cells were analyzed via an Amnis/Millipore FlowSight flow cytometer using an excitation wavelength of 642nm and emission detection between 642nm and 740nm.

### Annexin V/propidium iodide apoptosis assay

Cellular apoptosis was assessed via staining with fluorescently-labeled Annexin V and propidium iodide (PI) followed by flow cytometry analysis. Cells were treated in a manner identical to that described for the Alamar Blue assay with the exception of being plated at a density of 6x10^5^per well in 6-well cluster plates. On day 3, the cells were harvested using trypsin, washed with PBS, and resuspended in 50μl Annexin V binding buffer (BD Pharmingen). To the cell suspension, 5μl of FITC-conjugated Annexin V (BD Pharmingen) and 10μl of PI (BD Pharmingen) were added and allowed to incubate for 15 minutes at room temperature in the dark. Binding was assessed with a FlowSight flow cytometer (Amnis Corporation, EMD Millipore). FITCfluorochrome was excited using a 488nm laser with emission detection between 505 and 560nm. Propidium iodide was excited with a 642nm laser and emission detected between 642 and 740nm.

### Mitochondrial membrane potential assessment (TMRE)

Cells were incubated for 30 minutes with 100nM TMRE (tetramethylrhodamine, ethyl ester) (Sigma) before being centrifuged, washed once with PBS, resuspended in 40ul of PBS, and transferred to microcentrifuge tubes for FACS analysis. The cells were then analyzed via FACS (Flowsight, Amnis-Millipore) using a 488nm excitation laser. Intact and single cells were gated and the mean channel fluorescence of the gated cells was used to assess cells with active mitochondria.

### Western blotting

Cells were plated and treated as described above. On day 3, the spent culture media was carefully removed from the cells, and cells were washed twice with ice-cold PBS in situ, and 100μl of RIPA buffer containing protease inhibitor cocktail (Pierce) and PhosStop phosphatase inhibitor cocktail (Roche) was added. Cluster plates were then scraped and lysates were placed in tubes on ice for 30 min, with intermittentvortexing. Lysates were centrifuged at 13,000g for 20 minutes at 4°C and supernatants (soluble fraction) collected. A Bradford protein assay was performed to determine total protein concentration so that 30μg of total protein could be loaded into each well of a 4–15% Mini Protean TGX gel (Bio-Rad). The gels were run at 300V for approximately 15 minutes and then transferred onto a .2μm pore PVDF membrane (Bio-Rad) via 100V for 1 hour. Membranes were blocked with either 1% BSA in PBS or 5% skim milk in TBS-T for 1 hour and then incubated with primary antibodies in blocking buffer (1:1000) overnight at 4°C. Membranes were then washed 3 times for 5 minutes each with TBS-T and then incubated with HRP-conjugated secondary antibodies in blocking buffer (1:10,000) for 1 hour at room temperature. Membranes were then washed 3 times with TBS-T and bound antibody was detected via SuperSignalWest Pico Chemiluminescent Substrate (Thermo Scientific) and visualized using an ImageQuant LAS 4000 mini (GE Healthcare). Membranes were washed and re-probed for β-actin as a loading control. Analysis of densitometry was done with Image J and normalized to β-actin loading control.

### Detection of surface EGFR/HER-1 expression

MDA-MB-468 cells were plated in 12-well cluster plates and the following day were left untreated (control) or treated with lapatinib, Th1 cytokines, or both. Twenty-four hours later 50μM of Z-DEVD-FMK (Selleckchem) was added to some groups to suppress caspase 3 activity. All cells were harvested after 72 hours total culture time and stained with EGFR/HER-1 antibody conjugated to PE/Dazzel 594 (Biolegend). Flow cytometry was performed using a Flowcyte device (Amnis-Millipore) employing a488nm excitation laser and results analyzed via Ideas software suite.

### Lymphocyte functional assays

For allogeneic MLRs, Human peripheral blood mononuclear cells were obtained from healthy volunteers via leukapheresis and used in experiments after provision of informed written consent, and in accordance with the principals of the Declaration of Helsinki and NIH guidelines for human subjects, through protocols approved by the Institutional Review Boards of the Cleveland Clinic (08–957) and Kent State University (18–421). Blood products were separated into CD14^pos^ peripheral blood monocyte and lymphocyte-enriched fractions via countercurrent centrifugal elutriation as described previously [19] and maintained cryopreserved in liquid nitrogen until use. Dendritic cells were derived from monocytes that were thawed and incubated overnight in Macrophage SFM media (Gibco) and supplemented with GM-CSF (50ng/ml) and IL-4 (1000U). The next morning 1000U of IFN-γ was added to the culture and after 2 additional hours LPS (50ng/ml) was added. The cells were allowed to incubate for another 4 hours before being harvested, washed, and resuspended in RPMI supplemented with 5% human serum. Allogeneic lymphocyte-enriched fractions were thawed and brought up in identical culture medium. The cells were combined at a ratio of 20:1 (lymphocytes: dendritic cells) and plated in a 48-well cluster dish at 1ml total volume per well. The allogeneic co-culture was either treated with lapatinib (2 μM) or left untreated for control. After 72 hours the supernatants were collected and analyzed for IFN-γ via ELISA, and the cells were harvested and stained with FITC- anti-CD4 antibody (biolegend) and APC-anti-CD69 antibody (Biolegend). Surface expression was assessed via Flow cytometry using aFlowsightdevice (Amnis-Millipore).

For ELISPOT, cryopreserved, unfractionated total peripheral blood mononuclear cells (PBMCs) and IFN-γ ELISPOT kits were purchased from Cellular Technology Limited (C.T.L., Shaker Heights, OH). As per manufacturer’s recommendations, the PBMC’s were thawed and plated at a density of 200,000 cells per well (either in the presence or absence of 2μM lapatinib) with CEF-Class I Peptide Pool “Plus” (a mixture of common viral peptides; 10μl/well, C.T.L) or tetanus toxin (2μg/well, Sigma) as recall antigens, or incubated in media alone (control). The next day the cells were removed and the number of IFN-ɣ spot-forming cells were assessed as per kit instructions. All plates were read on an ImmunoSpot analyzer (C.T.L).

### Statistical analysis

To analyze the difference between treatment groups in the Alamar blue, trypan blue, TMRE, and Western blot assays treatment groups were normalized to a control of no treatment. A one-way ANOVA was used to determine if there was a significant difference between means and a Holm-Sidak test was used priori to compare the difference between individual groups. SigmaPlot software was used to run all statistical analyses. Treatment groups were considered significantly different if the *p* value was <0.05.

## Results

### Th1 cytokines work in concert with lapatinib to suppress cellular metabolism

**Fig 1:**
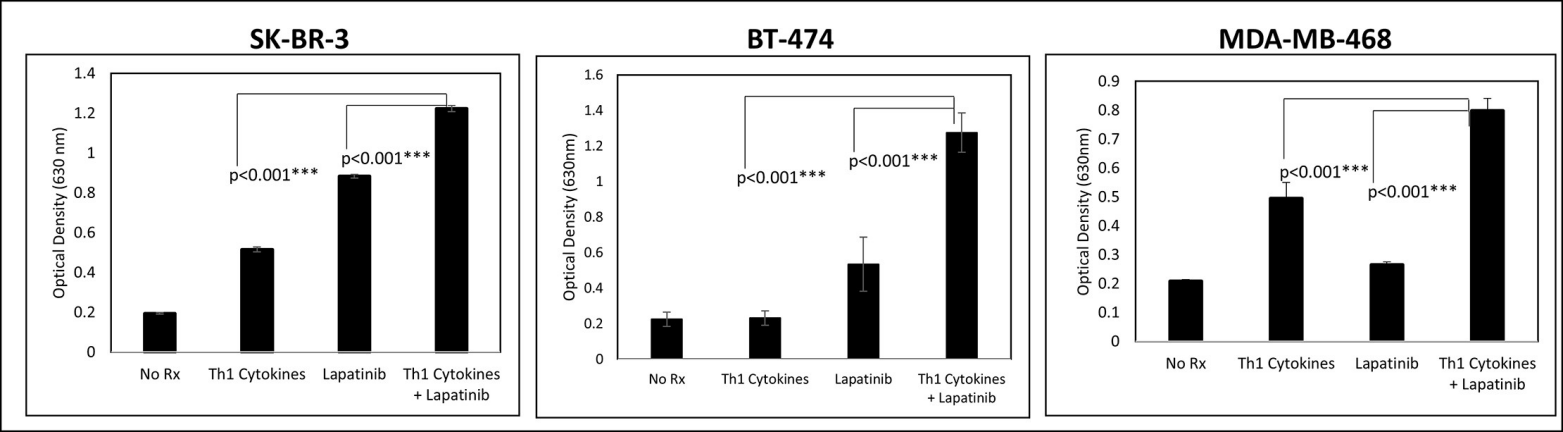
Lapatinib works in conjunction with Th1 cytokines to suppress metabolic activity of breast cancer cell lines. Cultured SK-BR-3, BT-474 and MDA-MB-468 cells were treated either with Th1 cytokines (TNF-α plus IFN-γ; 20 and 12.5ng/ml respectively), Lapatinib (200nM), Lapatinib plus Th1 cytokines or left untreated (control). After 72 hours incubation, resazurin sodium salt was added to each well and further incubated until color change was noted, and optical densities of supernatants read spectrophotometrically at 630nM. Shown are composite data from at least three trials per cell line expressed as mean optical density +/- SEM. Statistical significance was determined by one-way ANOVA followed by the Holm-Sidak multiple comparison test.

The anti-cancer drug lapatinib exerts its pharmacological effects by selectively interacting with the ATP-binding cassette of both HER-2 and EGFR, thereby interfering with transmembrane signaling [20]. Therefore, to determine whether Th1 cytokines could enhance lapatinib’s effect on breast cancer cells, we studied in detail three cell lines expressing one or more of these HER-family drug targets;SK-BR-3 (HER-2^hi^/EGFR^med^/ER^neg^), MDA-MB-468 (HER-2^neg^/EGFR^hi^/ER^neg^ and BT-474 (HER-2^hi^/EGFR^med^/ER^pos^).We began our studies with the Alamar Blue assay, which estimates levels of cellular metabolism via the reduction of resazurin salt to resofurin; a conversion that can be followed spectrophotometrically. Fig 1 displays optical densities of dye solution acted on by breast cancer cells that were either left untreated (control), incubated with Th1 cytokines alone (10ug/ml each), lapatinib alone (200nM), or both treatments. Lower optical density values indicate high metabolic activity (i.e. metabolism-dependent conversion of colored reactant to non-colored product). In all three cell lines, we observed significantly greater suppression of metabolic activity when cells were treated with lapatinib plus Th1 cytokines, compared with either treatment alone.

### Th1 cytokines work in concert with Lapatinib to induce cell death

**Fig 2:**
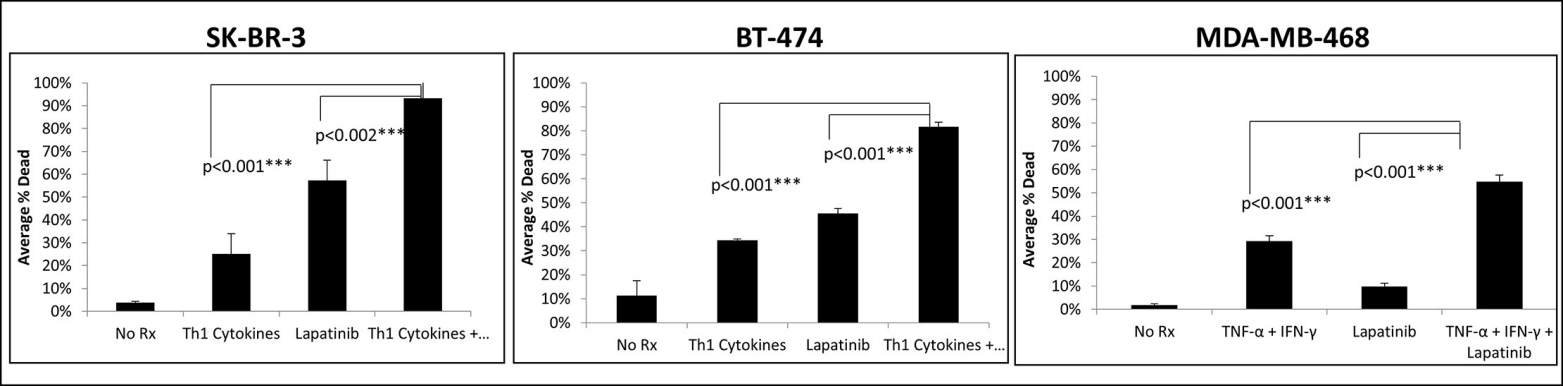
Lapatinib works in conjunction with Th1 cytokines to increase percentage of non-viable cells. Cultured SK-BR-3, BT-474 and MDA-MB-468 cells were treated either with Th1 cytokines (TNF-α plus IFN-γ; 20 and12.5ng/ml respectively), Lapatinib (200nM), Lapatinib plus Th1 cytokines or left untreated (control). After 72 hours, cells were harvested, stained with Trypan Blue dye and percentage of viable and non-viable cells enumerated microscopically with the aid of a hemocytometer. Shown are composite data from three independent experiments with each cell line expressed as the percentage stained (i.e. non-viable) cells of total cells counted. Statistical significance was determined by one-way ANOVA on percent dead followed by the Holm-Sidak multiple comparison test.

To determine if the lower relative cellular metabolism observed in the Alamar Blue assay was due to actual cell death, we used a vital staining technique based on Trypan Blue dye exclusion. Cells were either left untreated (control), or treated with Th1 cytokines alone, lapatinib alone, or lapatinib plus Th1 cytokines. After 3 days the cells were harvested and stained with Trypan Blue. The cells were then counted on a hemocytometer and categorized as alive if they did not stain blue or dead if they did. The results indicated that for all three cell lines tested, treatments were causing actual cell death, and that combined drug plus cytokine treatments caused significantly more cell death than either treatment alone (Fig 2).

### Cell death occurs via an apparent apoptotic mechanism

To determine whether the observed cell death occurred via an apoptotic mechanism, we employed two flow cytometry-based assays: TMRE staining and Annexin V/propidium iodide staining. The former is a fluorescent dye that is sequestered in healthy, metabolically-active mitochondria (providing a strong fluorescent signal), but diminished when these organelles lose their membrane potential as a consequence of apoptosis (weak fluorescent signal). The latter assay takes advantage of the ability of Annexin V to bind phosphoserine residues that are flipped to the outer leaf of the plasma membrane in early apoptosis, and the entry of propidium iodide into the nuclei and subsequent intercalation into DNA in late apoptotic cells that lose nuclear membrane integrity. Cells staining positive for both Annexin V and propidium iodide are therefore considered to be in late stage apoptosis.

**Fig 3:**
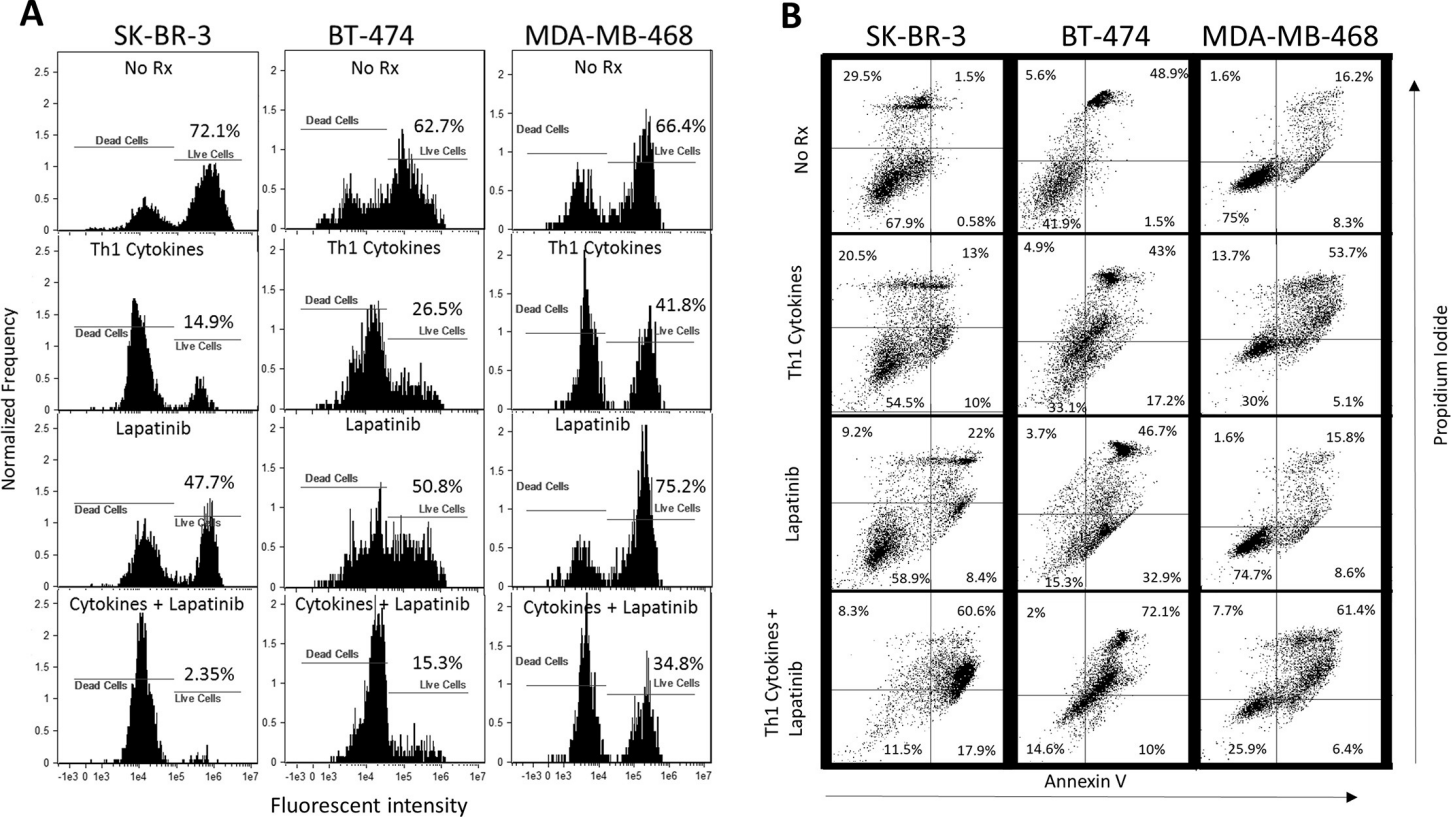
Lapatinib works in conjunction with Th1 cytokines to maximize indicators of apoptotic cell death. A) Cultured SK-BR-3, BT-474 and MDA-MB-468 cells were treated either with Th1 cytokines (TNF-α plus IFN-γ; 20 and 12.5ng/ml respectively), Lapatinib (200nM), Lapatinib plus Th1 cytokines or left untreated (control). After 72 hours incubation, cells were treated with 100nMtetramethylrhodamine ethyl ester (TMRE) dye to estimate mitochondrial ΔѰ, as an indicator of apoptotic cell death, as assessed by flow cytometry (lower fluorescent intensity indicates disruption of mitochondrial ΔѰ as a consequence of apoptosis). B) Identically cultured and treated cells were harvested and stained with FITC-Annexin V and propidium iodide (PI) and subjected to FACS analysis. Two-color dot-plot analysis displays percentage of gated cells in each quadrant (double-staining cells represent late apoptotic events).

For TMRE studies, whereas each cell line showed differences in absolute sensitivities to either cytokines alone, or lapatinib alone, all lines consistently showed the greatest loss in TMRE staining (indicating apoptosis) when the two treatments were combined (Fig 3A). For example, untreated SK-BR-3 showed 72.1% live cells, while cytokine-treated and lapatinib-treated groups demonstrated viabilities of 14.9% and 47.7%, respectively. In contrast, only 2.35% of the cells remained viable with cytokine plus lapatinib treatment.

For Annexin V/PI studies, stained, untreated SK-BR-3 cells showed that 1.5% were in stages of late apoptosis, while cytokine-treated and lapatinib-treated groups showed 13% and 22%, respectively (Fig 3B left panels). On the other hand, 60.6% of cells exposed to both treatments were in late stages of apoptosis. For BT-474 cells (Fig 3B middle panels), percentages of late apoptotic cells were 48.9% (untreated), 43% (cytokines), 46.7% (lapatinib) and 72.1% (both treatments). MDA-MD-468 cells had a baseline level of late apoptosis in the untreated group of 16.2% (Fig 3B right panels). These cells showed greater sensitivity for induced apoptosis by Th1 cytokines alone (53.7%), but were relatively less sensitive to lapatinb alone (15.8%) compared to the other cell lines. Nonetheless, the combination of Th1 cytokines andlapatinib was able to increase the percentage of cells in late stage apoptosis (61.4%) over single treatments. The results of this experiment confirm the additive effects of combined treatment and suggest that the induced cell death observed proceeds by an apoptotic mechanism.

### Th1 cytokines work in concert with Lapatinib to reduce levels of total and phosphorylated HER-family proteins

**Fig 4:**
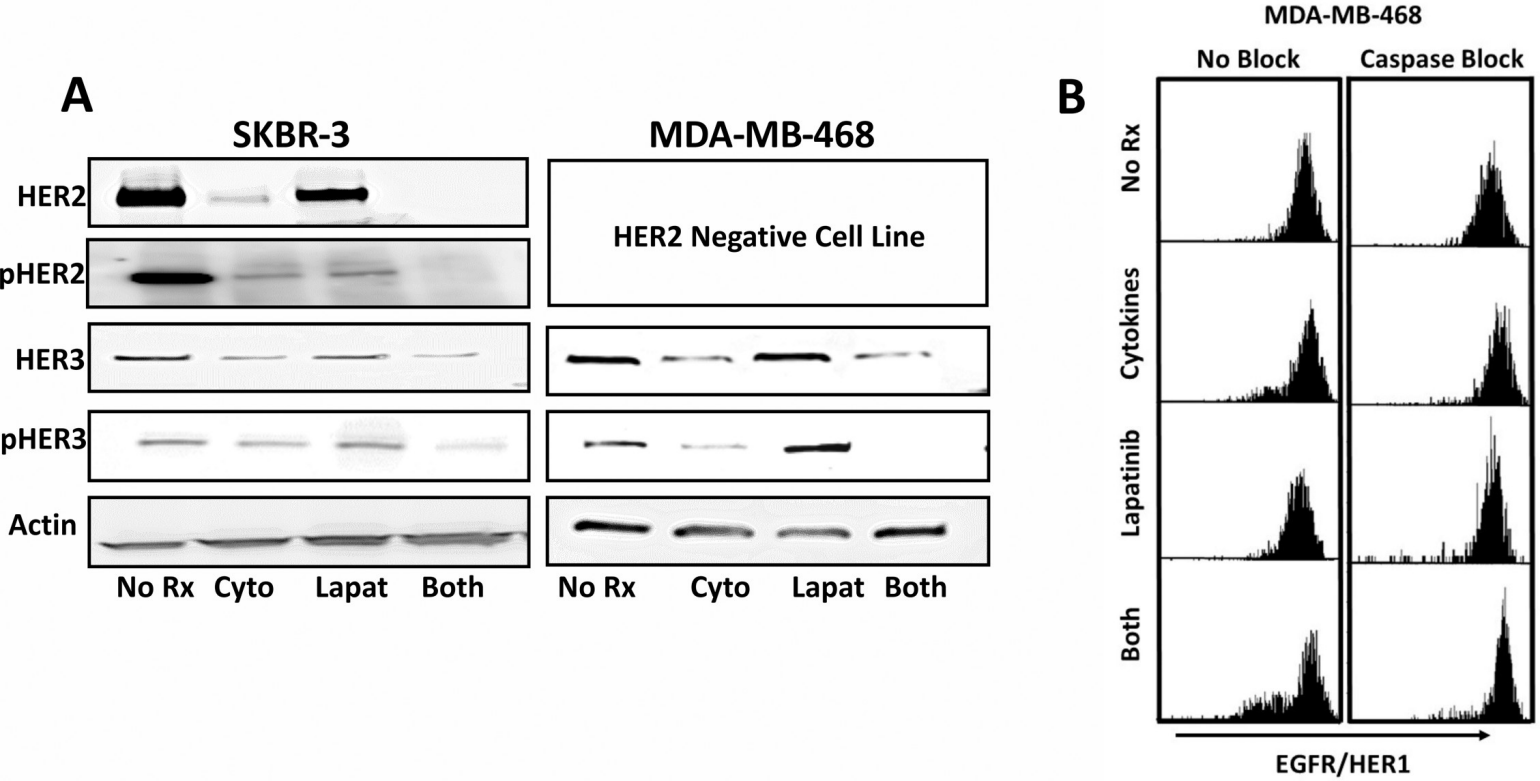
Lapatinib and Th1 cytokines suppress HER-family RTK expression and phosphorylation status. SK-BR-3 and MDA-MB-468 cells were treated with either Th1 cytokines (TNF-α plus IFN-γ; 20 and 12.5ng/ml respectively), 200nM Lapatinib, Th1 cytokines plus Lapatinib, or left untreated (control). A) After 72 hours incubation the cells were harvested, extracted in presence protease and phosphatase inhibitors, and 30μg/well total proteins separated on a 4–15% gradient SDS-PAGE gel prior to electrotransfer onto nitrocellulose membranes. Blots were then probed with anti-HER2, anti-phospho-HER2, anti-HER3 and anti-phospho-HER3 antibodies, and bands detected using chemiluminescence. B) After 24 hours, MDA-MB-468 cells treated as in (A) were supplied with caspase antagonist Z-DEVD-F. After 72 hours total culture cells were harvested and stained with EGFR antibody conjugated to PE/Dazzel 594 and analyzed for EGFR expression via flow cytometry.

To determine whether the enhanced apoptosis observed with combined treatment was reflected in alterations in the HER signaling pathway, we performed Western blot analysis of both HER-2 and HER-3 expression (both total and phosphorylated forms).HER-3 lacks a functional kinase domain and is therefore not a direct target of lapatinib [21]. However, when HER-3 dimerizes with either HER-2, or to a lesser extent EGFR, its catalytic tail is activated through cross-phosphorylation and becomes a potent oncogenic driver [22]. Thus by blocking the kinase domain of HER-2 and EGFR, the catalytic activity of HER-3 is also reduced [23]. We chose to focus these studies on two cell lines; SK-BR-3 because the dual treatment of Th1 cytokines and lapatinib had the most pronounced effects in all assays, and MDA-MB-468, because although they lack HER-2, they present high levels of HER-3 and EGFR. Interestingly, Th1 cytokines were able to downregulate total HER-2 by themselves, but when lapatinib was added to the cytokines, total HER-2 expression was suppressed to nearly undetectable levels, as was pHER-2 (Fig 4A). In addition,the regulation of HER-3 proved generally congruent for both cell lines. Th1 cytokines were able to down-regulate total HER-3 but not eliminate it. The addition of lapatinib did not enhance the observed down-regulation of total HER-3 by Th1 cytokines but it was able to nearly eliminate observed phosphorylation of HER-3. Results from these experiments show that Th1 cytokines and lapatinib work together to suppress HER signaling; Th1 cytokines by lowering the amount of total HER-2 and HER-3, and lapatinib by reducing the phosphorylation of the remaining HER-family molecules.

Because the MDA-MB-468 cells are considered “triple-negative”, we wanted to determine whether any changes occurred in the expression of EGFR/HER-1. This would help explain how the Th1 cytokines and lapatinib worked cooperatively in this HER-2 non-expressing cell line. We noticed a small degree of down-regulation induced by Th1 cytokines alone, but this was substantially increased when lapatinib was added (Fig 4B left panels). This combination therefore drove down EGFR/HER-1 thereby limiting the expression of this growth factor receptor on a population of these cells.Interestingly, when an inhibitor of executioner caspase 3 was added, EGFR/HER-1 expression loss was prevented (Fig 4B right panels).

#### Lapatinib-resistant breast cancer lines gain enhanced sensitivity to drug in the presence of Th1 cytokines

**Fig 5:**
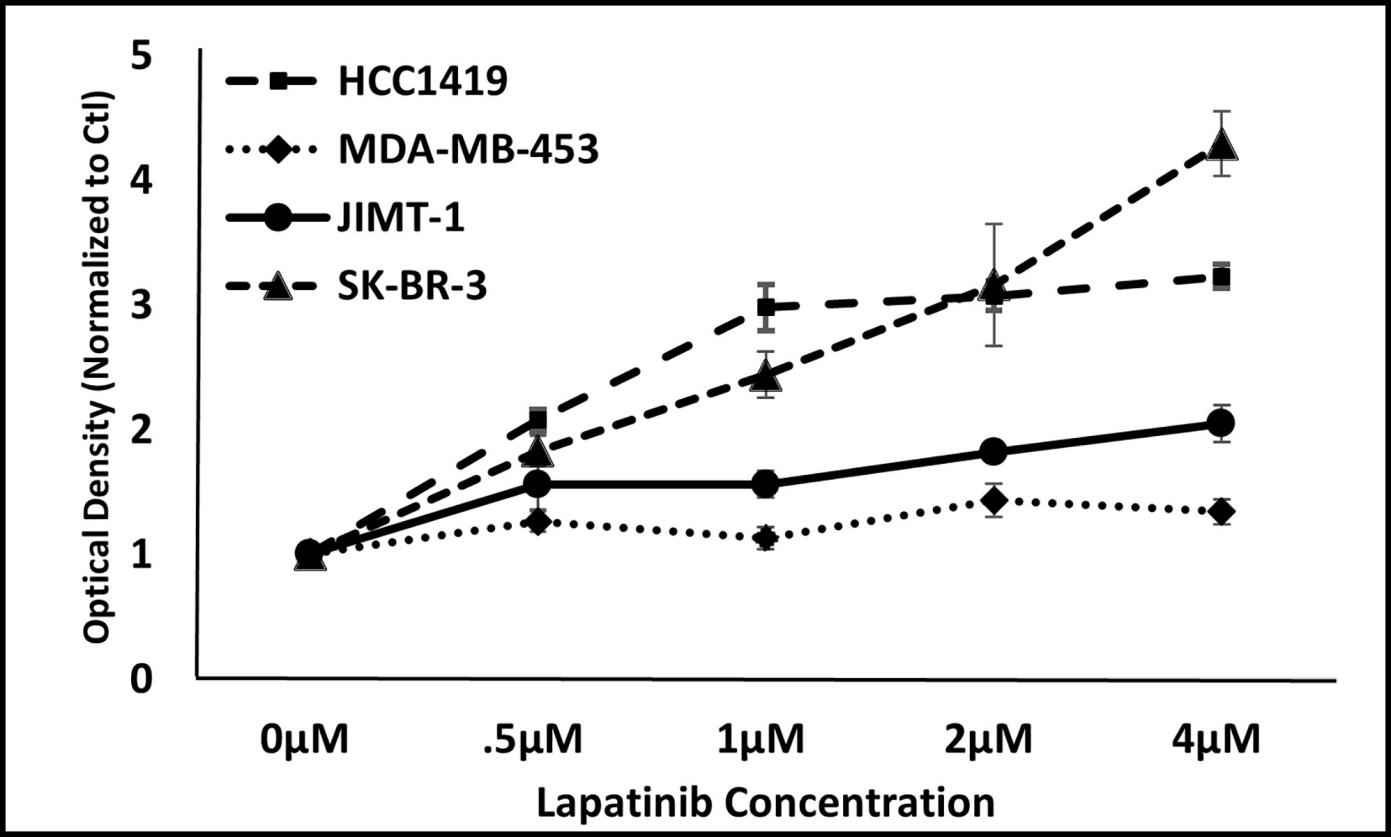
JIMT-1 and MDA-MB-453 cell lines display relative resistance to lapatinib. Cultured SK-BR-3, JIMT-1, HCC1419, and MDA-MB-453 cells were treated with increasing concentrations of lapatinib (0–4μM). After 72 hours incubation, resazurin sodium salt (Alamar Blue dye) was added to each well and further incubated until color change was noted, and optical densities of supernatants read spectrophotometrically at 630nM. Shown are composite data from at least three trials per cell line expressed as mean optical density +/- SEM.

We next investigated whether additional cell lines, which are known to be resistant to lapatinib, were nonetheless responsive to combinations of Th1 cytokine and drug.We chose to study JIMT-1 and MDA-MB-453 cells as examples of lapatinib-resistant lines [24]. For comparison, we also examined HCC1419 cells which are Herceptin-resistant but lapatinib-sensitive, as well as SK-BR-3 cells which are sensitive to both drugs [24, 25]. Our initial studies were designed to verify that these lines responded to lapatinib treatment as previous studies had reported. All lines were cultured in the presence of increasing doses of lapatinib (0.5–4μM) for 3 days and cellular metabolism assessed via the Alamar Blue assay (Fig 5). For the lapatinib-sensitive lines, the ability to metabolize Alamar Blue dye was strongly inhibited with increasing doses of lapatinib. In contrast, the resistant JIMT-1 and MDA-MB-453 cells, readily metabolized the dye even at maximal lapatinib concentrations, thereby verifying the previously-reported insensitivity of these two lines.

**Fig 6:**
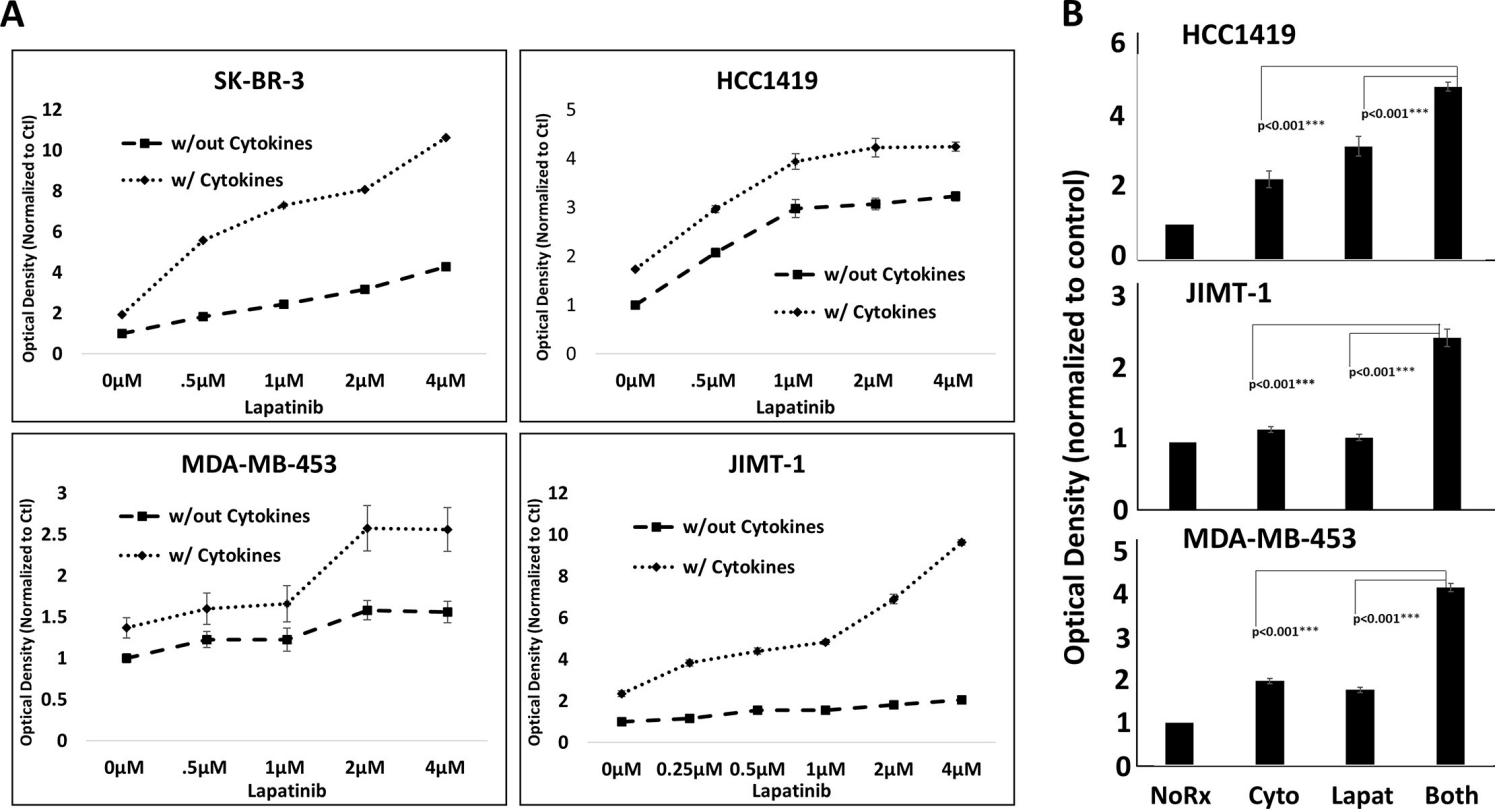
Lapatinib resistant lines are resensitized to drug in the presence of Th1 cytokines. A) Cultured SK-BR-3, HCC1419, JIMT-1, and MDA-MB-453 cells were treated either with Th1 cytokines (TNF-α plus IFN-γ; 20 and 12.5ng/ml respectively), Lapatinib (0–4μM), or Lapatinib plus Th1 cytokines. After 72 hours incubation, resazurin sodium salt was added to each well and further incubated until color change was noted, and optical densities of supernatants read spectrophotometrically at 630nM. B) Statistical analysis at 2μm concentration of lapatinib. Shown are composite data from at least three trials per cell line expressed as mean optical density +/- SEM. Statistical significance was determined by one-way ANOVA followed by the Holm-Sidak multiple comparison test.

We next examined lapatinib sensitivity in the presence or absence of a fixed concentration of Th1 cytokines (IFN-γ plus TNF-α). As expected, lapatinib plus Th1 cytokines worked cooperatively to suppress metabolic activity in the lapatinib-sensitive cell lines SK-Br-3 and HCC1419 (Fig 6A, upper panels). Surprisingly, cytokine-treated (but not untreated) JIMT-1 and MDA-MB-453 cells now displayed a dose-dependent sensitivity to lapatinib for suppression of cellular metabolism (Fig 6A lower panels). A statistical analysis at the 2μM lapatinib concentration demonstrating the significantly greater suppression in cellular metabolism with combined lapatinib plus Th1 cytokine treatment for the drug-resistant cells is shown in Fig 6B).

After demonstrating metabolic suppression, we turned our attention to the measurement of actual cell death. Trypan blue staining confirmed Alamar Blue results (S1 Fig), by demonstrating approximately equal sensitivity of HHC1419 cells to either Th1 cytokines or lapatinib. Maximum cell death was observed when both treatments were combined. On the other hand, JIMT-1 and MDA-MB-468 cells were relatively less sensitive to lapatinib as compared to Th1 cytokines alone, but when both treatments were combined, uptake of Trypan Blue dye increased significantly, with MDA-MB-468 cells showing particular synergy between Th1 cytokines and lapatinib.

**Fig 7:**
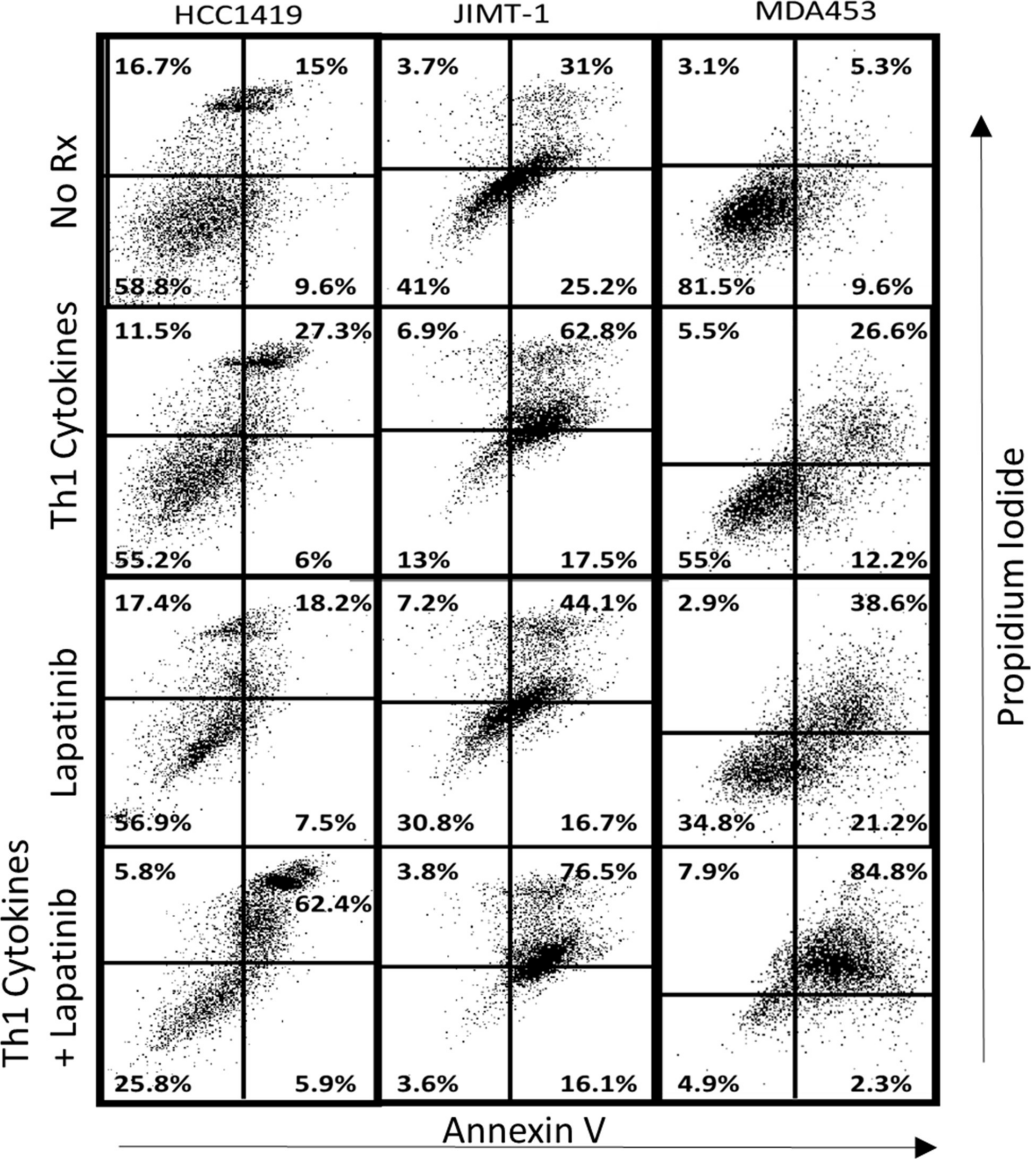
Lapatinib works in conjunction with Th1 cytokines to maximize indicators of apoptotic cell death in drug-resistant cell lines. Cultured HCC1419, JIMT-1 and MDA-MB-453 cells were treated either with Th1 cytokines (TNF-α plus IFN-γ; 20 and 12.5ng/ml respectively), Lapatinib (2μM), Lapatinib plus Th1 cytokines or left untreated (control). After 72 hours incubationcells were harvested and stained with FITC-Annexin V and propidium iodide (PI) and subjected to FACS analysis. Two-color dot-plot analysis displays percentage of gated cells in each quadrant (double-staining cells represent late apoptotic events).

We then looked for evidence that cell death occurred via an apoptotic mechanism using Annexin V/PI staining (Fig 7). JIMT-1 cells displayed an unusually high background of double-staining (apoptotic) cells (31%) which was increased to 62.8% by Th1 cytokine treatment, and a more modest 44.1% for lapatinib alone. When both treatments were combined, however, the percentage of late apoptotic cells increased to 76.5%. The results of MDA-MB-453 cells were even more dramatic, with untreated cells showing 5.3% double-positive, 26.6% with Th1 cytokines, 38.6% with lapatinib alone, but an impressive 84.8% late apoptotic cells when both treatments were combined.

### Lapatinib does not interfere with T cell function

**Fig 8:**
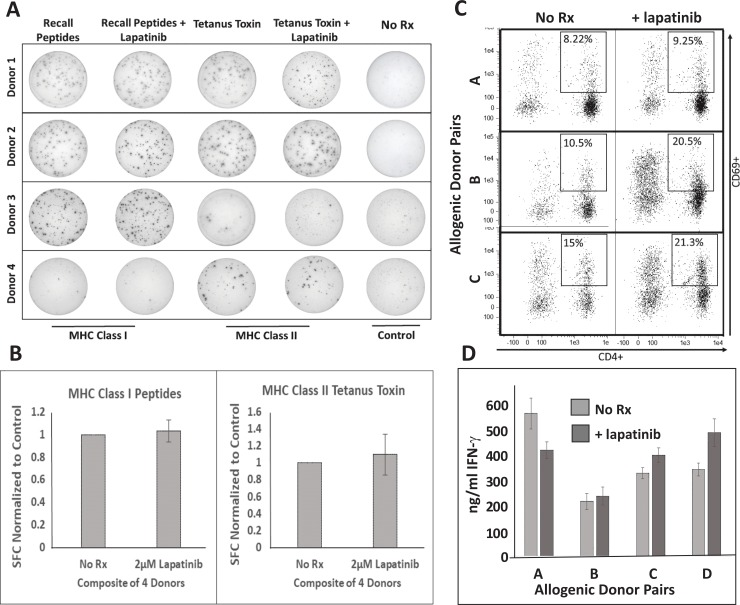
Lapatinib does not suppress T lymphocyte activation or function. A) IFN-γ ELISPOT assayof unfractionated human PBMCs from four unique donors stimulated with MHC class I recall peptides or tetanus toxoid protein in the presence of 2μM lapatinib. B) Composite data of the number of IFN-ɣ spot forming cells(normalized to control)from the same four donors. C) Flow cytometry analysis of allogenic DC:T cell co-cultures from three unique allogeneic stimulator:responder pairs with staining for CD4 (x axes) and CD69 (y axes). Percentage values indicate proportion of double-positive cells. D)ELISA analysis of IFN-ɣ production from four unique allogeneic stimulator:responder pairs in the presence or absence of lapatinib.

For lapatinib to be an effective companion drug for vaccine-based therapy, it must, as we have just demonstrated, work cooperatively with cytokine effectors of immunity to enhance killing of cancerous cells. However, it should also ideally have no deleterious effect on lymphocytes’ ability to become activated and produce these cytokines in the first place. Whereas lymphocytes are not generally regarded as major expressers of HER family members, reports of measurable, functional EGFR expression in some T cell subpopulations have been published [26]. We therefore conducted several standard assays of T lymphocyte activation and cytokine production in the presence or absence of lapatinib at doses identical to those used in these studies (Fig 8). We began with recall responses to common antigens against which we could expect a strong degree of pre-existing immunity, including a mixture of short synthetic peptides representing common viral antigens (MHC class I responses) as well as tetanus toxoid protein (presumably MHC class II responses). Interferon-ɣ ELISPOT analysis was performed on total PBMCs derived from four individual donors. One-way ANOVA analysis found no statistical difference in spot-forming cells for any antigen between lapatinib-treated and untreated groups. Example ELISPOT wells are shown in Fig 8A with composite results from the 4 donors illustrated in Fig 8B. We next examined T cell responses in the allogeneic MLR. Here, activated human monocyte-derived dendritic cells were co-cultured with MHC-unmatched lymphocytes derived from different donors in the presence or absence of lapatinib. After 72 hours of co-culture, both cells and culture supernatants were harvested and subjected to analysis. Harvested cells were stained for CD4 (to identify helper T cells), and CD69 (a T cell activation marker). In three separate DC:Lymphocyte allogenic pairings, no lapatinib-induced suppression was observed in the percentage of activated CD4^pos^cells (Fig 8C). To the contrary, lapatinib appeared to somewhat enhance the expression of the CD69 activation marker. Likewise, assessment of IFN-ɣ levels in allogeneic co-culture supernatants did not reveal suppression of IFN-ɣ production. We therefore conclude that lapatinib is unlikely to significantly interfere with T cell function.

## Discussion

Dendritic cell-based vaccines that target the HER-2 protooncogene and induce strong Th1 immunity can induce complete pathological responses in up to one third of all HER-2^pos^ ductal carcinoma in situ patients when supplied in the neoadjuvant setting [4, 7]. However, this response rate drops precipitously after the cancer becomes invasive [7]. This means that in order to improve response rates both for DCIS patients as well as individuals with more advanced cancer, vaccination will likely need to be paired with other treatment modalities such as drug or radiation therapy.

Evidence has accumulated that Th1 cells, and in particular the cytokines they produce, may be instrumental in controlling some malignancies through the induction of cellular senescence or apoptosis [6, 27]. In our hands, the combination of IFN-γ and TNF-α could mimic, on cultured breast cancer lines, many of the effects observed in vaccinated individuals including cell death and suppression of HER-family oncodriver expression [6]. Furthermore, we showed that addition of anti-estrogen drugs to cultured ER^pos^ breast cancer lines could greatly enhance cell death when combined with Th1 cytokines [7]. This finding was exploited in a recent clinical trial where patients with ER^pos^/HER-2^pos^ DCIS were given a short course of anti-estrogen drugs coincident with anti-HER-2 vaccination [7]. Complete response rates of this patient subgroup jumped to nearly 30% from the previous response rate of 5% when anti-estrogen drugs were not given. This finding validated the concept that lower-toxicity targeted drugs could greatly enhance vaccine performance, as well as the practice of selecting such companion drugs using an in vitro screen that looks for strong enhancement by the putative pharmacological agent of the apoptosis-inducing effects of Th1 cytokines.

We therefore focused our attention on the small molecule drug, lapatinib. The HER family of RTKs require cross-phosphorylation by their dimerization partner for activation [28–35]. Lapatinib selectively blocks cross phosphorylation in EGFR and HER-2 by binding to the ATP binding cassette of the kinase domain [8, 9]. HER-3, lacking a kinase domain of its own, is dependent on a dimerization partner for phosphorylation and subsequent activation [21]. EGFR and HER-2 are both known to act as dimerization partners for HER-3 [36–38]; therefore blocking phosphorylation of both HER-2 and EGFR minimizes phosphorylation opportunities for HER-3 [23]. Moreover, the HER-2/HER-3 heterodimer signaling cascade, known for its potent oncogenicity [22], is rendered inactive. Because our previous studies showed that Th1 cytokines could drive down both HER-2 and HER-3 expression on multiple breast cancer cell lines [6], we hypothesized that Th1 cytokines and lapatinib would work well together; cytokines to diminish total HER expression, and lapatinib to block the activation of the remaining expressed HER family RTKs. Such a double-hit could in theory virtually eliminate the downstream signaling events critical for maintenance of malignant phenotype including continued cell proliferation and resistance to apoptosis.

We began by examining three breast cancer cell lines, Sk-Br-3, BT-474 and MDA-MB-468. We found that by all examined criteria, including cellular metabolic activity (Alamar Blue assay), observed percentage of dead cells (Trypan Blue Staining) and assessment of cellular apoptosis (Annexin V/PI staining), the combination of Th1 cytokines and lapatinib acted in concert to maximize the negative impact on the tumor cells. Western Blot analysis of levels of HER-2 and HER-3 (both total and phosphorylated forms) confirmed our original hypothesis: The combination of Th1 cytokines and lapatinib reduced phosphorylated forms of HER-2 and HER-3 to nearly undetectable levels, with the cytokines chiefly responsible for driving down total HER expression and lapatinib mostly preventing phosphorylation (Fig 4A).When we looked at EGFR/HER-1 expression by FACS analysis, we found that Th1 cytokines and lapatinib also worked cooperatively to drive down the surface expression of this growth factor receptor (Fig 4B). It was interesting to note that the caspase inhibitor, Z-DEVD-FMK prevented this down-regulation. This suggests that the down-regulation of EGFR/HER-1is not helping to initiate the process of apoptosis, but instead the down-regulation is a result of progressive apoptosis. A reasonable explanation for this is that as cells undergo apoptosis, the presence of growth factor receptors or other downstream promotors of cell division and survival supply competing signals that may interfere with the apoptotic process. It may be that certain critical promotors of cell survival and proliferation are especially sensitive to the action of caspases so that once apoptosis has proceeded past a certain critical point, these components are selectively eliminated to speed the process of cell death. Our laboratory has noted that additional such targets, under the influence of Th1 cytokines, are suppressed including Akt kinase, while others, such as kras appear untouched (manuscripts in preparation).

The promise of targeted drug therapy stems from the opportunity for greater effectiveness, and in particular, fewer side effects because the malignant cells are much more dependent upon the drug target than normal, healthy tissues. Nonetheless, this selectivity is not absolute, and overall effectiveness for most targeted drugs is still circumscribed by dose-limiting toxicities. This may be particularly the case when more than one targeted drug is employed at the same time [39]. The pairing of targeted drugs with immunization that stimulates strong Th1 immunity, however, offers some attractive possibilities. We showed in previous studies that HER-2^pos^ DCIS patients vaccinated with IL-12-secreting dendritic cells pulsed with HER-2 peptides not only generated strong Th1 immune responses evident in peripheral blood, but also developed heavy infiltrates of both CD4^pos^ T cells as well as B lymphocytes into the area of disease in the breast [3]. Under these circumstances it is likely that the MHC class II–positive B cells can serve as antigen-presenting cells for the Th1 cells, and that T cells, so activated, can secrete Th1 cytokines into the tumor beds. These cytokine-secreting T cells, unlike traditional drugs, concentrate at the site of disease to disgorge their cargo of senescence- and apoptosis-inducing factors. The present studies show that in an in vitro system, Th1 cytokines plus the targeted drug, lapatinib, work together to enhance tumor kill, making the combination of lapatinib plus DC-based vaccination against HER-2 an attractive combination to test in the near future for HER-2^pos^ breast disease. It should also be noted that the combination of Th1 cytokines and lapatinib potentiated cell death in the triple-negative cell line MDA-MB-468 as well, suggesting that targeting this phenotype with Th1-polarizing immunotherapies plus lapatinib may be a fruitful endeavor.

Our demonstration in vitro, that lapatinib does not interfere with T cell function (Fig 8), further recommends the possible use of this drug or those like it in combination with active immune-based therapy. In fact, we found the percentage of CD69^pos^ (i.e. activated) CD4 T cells appeared to be somewhat increased in the presence of lapatinib for the allogeneic MLR. This unexpected enhancement may be explained by the recent report that regulatory T cells can be controlled through the EGFR signaling axis, and furthermore the EGFR/HER-1-selective inhibitor drug gefitinib could attenuate T_reg_ function [40]. Since lapatinib and gefitinib both inhibit EGFR/HER-1, lapatinib and drugs like it could actually enhance immune responses at least in part by lowering T_reg_ activity-making them inherently compatible for combination therapy with immune-based approaches.

The studies also suggest another avenue of investigation; the possibility that having cytokine-secreting Th1 cells trafficking to the site of disease could locally sensitize the tumor cells to targeted drug action such that these agents, which could be added singly or in combinations, could be administered at lower doses that would be required if only drugs were being used. In this way tumor beds would be selectively sensitized for drug activity leaving normal, distal tissues not infiltrated by lymphocytes less affected by the drugs. Our in vitro data shows that the presence of Th1 cytokines can lower by several fold the amount of lapatinib necessary to achieve the same level of tumor killing. It would be highly advantageous if Th1 vaccination could not only enhance the clinical activity of targeted drugs, but also allow them to be used at lower doses to decrease toxicity. This possibility that this will be the case in vivo is currently being investigated in our laboratory.

Targeted anti-cancer drugs for breast and other malignancies hold great promise for revolutionizing the way these diseases are treated, but besides dose-limiting toxicity, another overarching limitation has been the rapid development of drug resistance, even after robust initial clinical responses. This is true not only for HER-2-targeted drugs [41, 42], but also for drugs directed against BRAF and MAP kinase pathways [43], EGFR [44] and others. An unanticipated finding in this study was the observation that lapatinib-resistant cell lines JIMT-1 and MDA-MB-453 demonstrated enhanced drug sensitivity in the presence of Th1 cytokines. This finding indicates that Th1 cytokines are, through some currently unknown mechanism, capable of circumventing at least some mechanisms of lapatinib (and perhaps other drug) resistance. It also is suggestive that Th1-polarizing vaccines given in combination with targeted therapies may create an environment where tumor cells cannot efficiently express certain drug-resistant phenotypes, thereby increasing the overall effectiveness of therapy. The mechanisms whereby re-sensitization to lapatinib occurs under the influence of Th1 cytokines (and whether this effect is seen in resistance to other targeted drugs) is currently being explored in our laboratory.

In summary, these findings demonstrate that the small molecule RTK antagonist drug, lapainib, works in conjunction with Th1 cytokines to maximize induced cell death in a variety of lapatinib-sensitive and lapatinib-resistant breast cancer cell lines. This provides justification for additional clinical trials that will include the pairing of lapatinib with anti-HER-2 DC1 immunization to enhance responsiveness to vaccine therapy, and furthermore encourages the continued search for additional agents that work cooperatively with Th1 cytokines to enhance tumor killing. The greatest opportunities for leveraging vaccine efficacy may lie through combinations of targeted drugs that interfere with growth factor signaling or regulation of cell cycle.

## Supporting information

S1 Fig Lapatinib works in conjunction with Th1 cytokines to increase cell death. Cultured HCC1419, JIMT-1, and MDA-MB 453 cells were treated with lapatinib (2μM), Th1 cytokines (TNF-α plus IFN-γ; 20 and 12.5ng/ml respectively), lapatinib plus Th1 cytokines, or left untreated for control. After 72 hours incubation the cells were harvested, subjected to .002% Trypan Blue dye, and the mean fluorescence (ex 642nm) was read via flow cytometry. Shown are composite data of a minimum of three trials per cell line normalized to control +/- SEM. Statistical significance was determined by one-way ANOVA followed by the Holm-Sidak multiple comparison test
(PDF)
