# Supplementary figures and images for: Th1 cytokines sensitize HER-expressing breast cancer cells to lapatinib
Source: PLoS One. 2019 Jan 18;14(1):e0210209. doi: 10.1371/journal.pone.0210209 (PMC6338365; doi:10.1371/journal.pone.0210209)

## Supplemental Figure

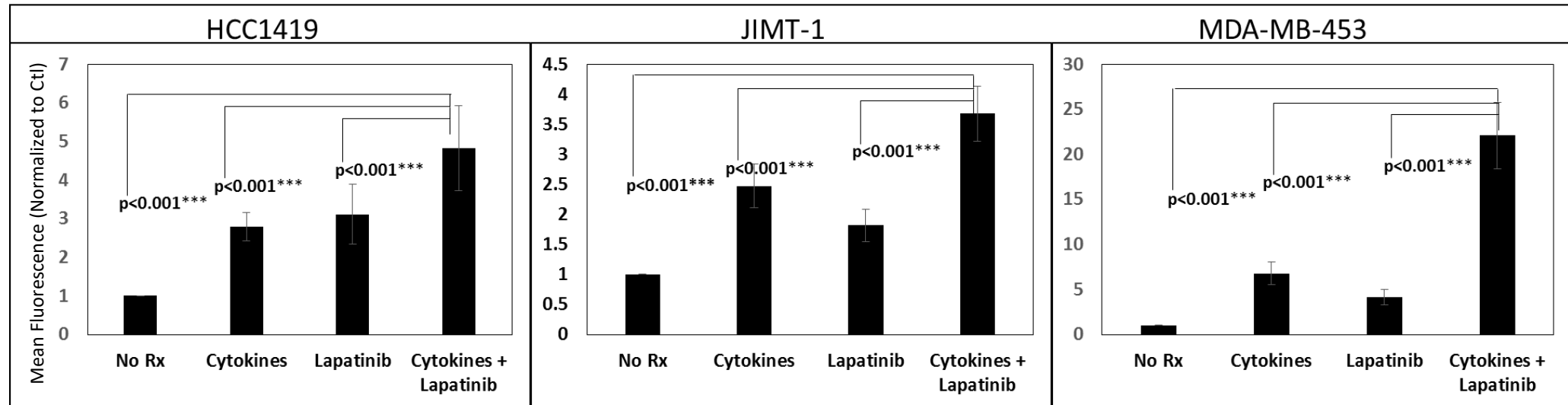

Supplement: S1 Fig — Cultured HCC1419, JIMT-1, and MDA-MB 453 cells were treated with lapatinib (2μM), Th1 cytokines (TNF-α plus IFN-γ; 20 and 12.5ng/ml respectively), lapatinib plus Th1 cytokines, or left untreated for control. After 72 hours incubation the cells were harvested, subjected to .002% Trypan Blue dye, and the mean fluorescence (ex 642nm) was read via flow cytometry. Shown are composite data of a minimum of three trials per cell line normalized to control +/- SEM. Statistical significance was determined by one-way ANOVA followed by the Holm-Sidak multiple comparison test (PDF) [file pone.0210209.s001.pdf]
